# Chemogenetic Silencing of Prelimbic Cortex to Anterior Dorsomedial Striatum Projection Attenuates Operant Responding

**DOI:** 10.1523/ENEURO.0125-19.2019

**Published:** 2019-10-18

**Authors:** Megan L. Shipman, Gregory C. Johnson, Mark E. Bouton, John T. Green

**Affiliations:** 1 Department of Psychological Science; 2 Neuroscience Graduate Program, University of Vermont, Burlington, VT 05405

**Keywords:** action, dorsomedial striatum, DREADD, instrumental conditioning, operant conditioning, prelimbic cortex

## Abstract

Operant (instrumental) conditioning is a laboratory analog for voluntary behavior and involves learning to make a response for a reinforcing outcome. The prelimbic cortex (PL), a region of the rodent medial prefrontal cortex, and the dorsomedial striatum (DMS), have been separately established as important in the acquisition of minimally-trained operant behavior. Despite dense anatomical connections between the two regions, experimenters have only recently linked projections from the PL to the posterior DMS (pDMS) in the acquisition of an operant response. Yet, it is still unknown if these projections mediate behavioral expression, and if more anterior regions of the DMS (aDMS), which receive dense projections from the PL, are also involved. Therefore, we utilized designer receptors exclusively activated by designer drugs (DREADDs) to test whether or not projections from the PL to the aDMS influence the expression of operant behavior. Rats underwent bilateral PL-targeted infusions of either a DREADD virus (AAV8-hSyn-hM4D(Gi)-mCherry) or a control virus (AAV8-hSyn-GFP). In addition, guide cannulae were implanted bilaterally in the aDMS. Rats were tested with both clozapine-N-oxide (CNO) (DREADD ligand) and vehicle infusions into the aDMS. Animals that had received the DREADD virus, but not the control virus, showed attenuated responding when they received CNO microinfusions into the aDMS, compared to vehicle infusions. Patch clamp electrophysiology verified the inhibitory effect of CNO on DREADDs-expressing PL neurons in acute brain slices. GFP-expressing control PL neurons were unaffected by CNO. The results add to the recent literature suggesting that connections between the PL and aDMS are important for the expression of minimally-trained operant responding.

## Significance Statement

Only very recently has it been shown that prelimbic cortex (PL) projections to the posterior dorsomedial striatum (DMS) are important in the acquisition of operant responding. Here, we show that PL projections to the anterior DMS (aDMS) are important in the expression of operant responding.

## Introduction

The prelimbic cortex (PL) has been well established as a mediator of operant (instrumental) responses early in training (Corbit and Balleine, 2003; Killcross and Coutureau, 2003; Ostlund and Balleine, 2005; Tran-Tu-Yen et al., 2009; Trask et al., 2017; Shipman et al., 2018). The dorsomedial striatum (DMS) has similarly been implicated in the acquisition and expression of operant responding, with a particular emphasis on the posterior DMS (pDMS; Yin et al., 2005a,b; Shiflett et al., 2010). Because the PL and pDMS have both been implicated in the early acquisition of operant responding, it has been suggested that they may function together as part of a greater circuit supporting goal-directed operant responding (Corbit, 2018). Indeed, lesion disconnection of these two regions before acquisition sessions disrupts the expression of operant responding at test (Hart et al., 2018a).

Traditional disconnection studies do not address the question of whether or not function is mediated by a direct versus an indirect connection between two brain regions. Recent research using designer receptors exclusively activated by designer drugs (DREADDs) has shown that PL to pDMS projections are important for the acquisition of operant responding (Hart et al., 2018b). Hart, Balleine, and colleagues used a dual-virus approach to inactivate the PL-pDMS pathway by infusing AAV-Cre recombinase into the pDMS, and a Cre-dependent DREADDs viral construct into the PL. They found that silencing the PL-pDMS pathway during acquisition, via systemic injection of the DREADDs ligand clozapine-N-oxide (CNO), reduced operant responding during test (Hart et al., 2018b).

The PL has been implicated in the expression of minimally-trained operant responding when testing occurs in the acquisition context (Trask et al., 2017; Shipman et al., 2018). Temporary inactivation of the PL with baclofen/muscimol at the time of test following six daily sessions of acquisition (lever press training) resulted in an attenuation of operant responding in the context where training had been conducted, but not in another context (Trask et al., 2017). Hart et al. (2018b) showed that PL projections to pDMS are important in the acquisition of operant behavior, but they did not examine whether PL projections to the DMS are important for the expression of operant behavior. In addition, Hart et al., did not examine the function of PL projections to the anterior DMS (aDMS); some studies suggest that PL projections to the aDMS are at least as dense as PL projections to pDMS (Mailly et al., 2013; Hunnicutt et al., 2016).

In the current experiment, we hypothesized that PL projections to the aDMS are involved in the expression of operant responding in the acquisition context. Six weeks before training, we infused an AAV8-DREADDs or control viral construct bilaterally into the PL and implanted bilateral guide cannulae into the aDMS. Rats underwent 6 d of instrumental conditioning followed by infusion of CNO or vehicle into the aDMS before test. We found that silencing projections from the PL to a relatively anterior region of the DMS attenuated lever-press responding, implicating this pathway in the expression of operant responding. Patch-clamp electrophysiology in a separate group of rats confirmed that CNO suppressed spiking in DREADDs-expressing, layer 5 PL pyramidal neurons but not in PL neurons that expressed the control, GFP, construct.

## Materials and Methods

All animal procedures were performed in accordance with the University of Vermont animal care committee’s regulations.

### Subjects

The subjects were 24 male Wistar rats from Charles River Laboratories. Rats were 59–63 d old and initially housed in pairs on arrival. They were given at least 3 d to acclimate to the colony before undergoing surgery. Following surgery, rats were housed individually in a room maintained on a 12/12 h light/dark cycle. Experimentation occurred during the light portion of the cycle.

### Surgery

Rats were anaesthetized with isoflurane. pAAV8-hSyn-hM4D(Gi)-mCherry viral construct (gift from Bryan Roth; Addgene plasmid #50475; http://n2t.net/addgene:50475; RRID:Addgene_50475) or the control pAAV8-hSyn-EGFP viral construct (gift from Bryan Roth; Addgene plasmid #50465; http://n2t.net/addgene:50465; RRID:Addgene_50465) was infused bilaterally into the PL with a Hamilton syringe (stereotaxic coordinates AP: +3.0, ML: ±0.75, DV: –4.0) at a rate of 0.1 μl/min. Each side received an infusion of 0.8 μl. The needle was in place for 2 min before the start of the infusion to allow the brain to settle, and 10 min following completion of the infusion to allow for diffusion away from the needle tip. Guide cannulae (22 gauge, Plastics One) were targeted bilaterally to the aDMS at stereotaxic coordinates AP: +1.0, ML: ±2.0, DV: –3.6. Rats were given carprofen (5.0 mg/kg) for analgesia, as well as bupivacaine around the scalp incision, and Ringer’s solution (1.0 ml) following surgery. A second dose of carprofen was administered the following day. Rats were weighed and reduced to 90% free feeding weight 4 d before magazine training, and were maintained at 90% free feeding weight throughout the experiment.

### Apparatus

Two sets of four operant chambers were used for this experiment (Med Associates model ENV-008-VP). The sets were separated by room and differed slightly in their features. Differentiation of contexts was not required for this experiment, but rats were counterbalanced on vector type and the contexts where they received training/testing. Operant chambers measured 30.5 × 24.1 × 21.0 cm (l × w × h) and the food cup (measuring 5.1 × 5.1 cm) was located within the center of the front wall at a height of 2.5 cm above the floor. All chambers also featured a lever to the left of the magazine (Med Associates model ENV-112CM) that was inserted following a timeout period of 2 min at the beginning of each session. Within each room, each of the four chambers was housed in a sound attenuation chamber. Each chamber was lit by a single incandescent bulb (7.5 W) located on the sound attenuation chamber ceiling. Ventilation fans provided white noise (65 dBA).

Half the operant chambers featured clear, acrylic plastic on the walls and a ceiling with brushed aluminum on the front and rear walls. Floor panels were stainless steel grids (0.48 cm in diameter) that were staggered so that every other bar was in the opposite of two planes from the previous bar (one plane was 0.5 cm above the other). The other half of the chambers had all floor grids mounted in the same plane with each bar spaced 1.6 cm from the previous bar. The walls in these boxes were also acrylic plastic but featured black, diagonal stripes that were 3.8 cm wide and 3.8 cm apart.

The reinforcer used for this experiment was a 45-mg sucrose pellet (5-TUT:1811251, TestDiet). The pellet was delivered to the magazine by instruction through a computer located in an adjacent room.

### Procedure

All behavioral procedures were conducted so that both tests occurred six to seven weeks following vector infusion. Rats were run in cohorts of four or eight and counterbalanced across conditions.

### Magazine training

All rats received one half-hour session of magazine training. Once all animals were placed in their respective operant chambers, a 2-min timeout period began. During this period, no reinforcers were available. Following that, sucrose reinforcers were freely delivered to the food magazine on a RT 30 schedule. No levers were present during this training.

### Acquisition training

Rats then received six daily acquisition sessions. At the start of each session, once all rats were in their respective operant chambers, left levers were inserted into boxes after 2 min, and rats were reinforced on a VI-30 schedule for lever presses. Levers retracted following completion of the half-hour session. If rats initially failed to eat sucrose pellets, levers were baited with mashed pellets. One rat had to be removed from further analysis because it failed to eat any pellets and thus failed to acquire the operant lever-pressing response.

### Test

After acquisition, all rats underwent two test sessions, separated by a day of retraining. Before the first test session, half the rats received a 0.5-μl bilateral intracranial infusion of CNO (1.0 mM) and the other half received a vehicle infusion [artificial CSF (ACSF)] into the DMS (see slice preparation section for more specifics about ACSF composition). The CNO concentration of 1 mM was based on previous studies (Mahler et al., 2014; Lichtenberg et al., 2017). For infusions, dummy cannulae were removed and internal cannulae were inserted into guide cannulae. Internal cannulae tips protruded 1 mm below the tip of guide cannulae. Infusions were delivered over 2 min (0.25 μl/min) by internal cannulae attached to tubing (Intramedic) that connected to Hamilton syringes driven by a microinfusion pump (Kd Scientific). Internal cannulae were allowed to remain in place for 1 min following infusions before removal and reinsertion of dummy cannulae. Rats were then placed in transport containers and put into operant chambers 5–15 min after the infusion. After a 2-min period, levers were inserted into the operant chambers (as usual). The test ran for 10 min; lever press responses had no scheduled consequences (i.e., the test was conducted in extinction). The following day, rats received a session of retraining with the VI-30 reinforcement schedule. A second test was given the day after, in which rats received the opposite infusion of the first test. Other than receiving the opposite infusate, testing proceeded exactly as on the first test day.

### Histology

Following the second test, rats were injected with a lethal dose of sodium pentobarbital (150 mg/kg, i.p.) and transcardially perfused with PBS followed by 4% paraformaldehyde. Brains were removed and postfixed for 1 h before being transferred to a 30% sucrose/PBS solution. After sinking, brains were embedded in OCT and flash-frozen in 2-methylbutane that had been cooled with dry ice. The PL and DMS of each brain were sectioned at 60 μm and floated in phosphate buffer onto slides. Sections were dried in the dark before being mounted with Vectashield mounting medium with DAPI and coverslipped. Viral transfection was examined using a confocal microscope (Nikon C-2). Excitation lasers were 405 nm (DAPI), 488 nm (EGFP), and 561 nm (mCherry). Viral expression was examined for accuracy by comparing the location of PL cell expression to the PL location in a rat brain atlas (Paxinos and Watson, 2014). Axon terminals were examined for expression directly underneath the deepest part of the cannulae, which were confirmed to be in the DMS.

### Slice preparation for electrophysiology

Adult Wistar rats, of the same age and from the same supplier as above, were used for patch clamp electrophysiology. Rats underwent PL infusion of viral construct AAV8-hSyn-hM4D(Gi)-mCherry or AAV8-hSyn-EGFP as described above. Following at least six weeks of recovery, electrophysiology experiments were performed. On the experimental day, rats were deeply anesthetized with sodium pentobarbital and transcardially perfused with cold, sucrose-replaced artificial cerebrospinal fluid. The brain was then quickly removed and sliced in the coronal plane on a Leica VT1000S (Leica Instruments) vibratome. Brain slices were then allowed to recover in warmed sucrose-replaced artificial cerebrospinal fluid at 32°C for 30 min, and then equilibrated in room temperature ACSF for at least 30 min before recording. ACSF was composed of the following: 124 mM NaCl, 2.8 mM KCl, 2 mM CaCl, 1.25 mM NaH_2_PO_4_, 10 mM glucose, 0.4 mM sodium ascorbate, 2 mM sodium pyruvate, 2 mM MgSO_4_, and 26 mM NaHCO_3_. Sucrose-replaced ACSF was similar to recording-ACSF with the following exceptions: 0 mM NaCl, 206 mM sucrose, 1 mM CaCl, and 1 mM MgCl. Each was pH adjusted to 7.3–7.4 with HCl and osmolarity was 310 ± 5 mOsM.

### Recording procedures

Slices were transferred to a recording chamber (Warner Instruments) and continuously perfused with oxygenated, 32°C ACSF at a rate of 3–4 ml/min. Virally-infected cells were identified under fluorescent illumination in layer 5 of the PL using a Leica DM-LFSA microscope and Rolera Bolt 3000 CCD camera. Cells were then patched under brightfield/infrared illumination in current clamp mode. Electrodes were made from thin-walled borosilicate glass capillaries (World Precision Instruments) and pulled on a Sutter P-97 micropipette puller and filled with a K-glu intracellular solution composed of the following: 140 mM potassium gluconate, 2 mM KCl, 3 mM MgCl, 10 mM HEPES, 5 mM phosphocreatine, 2 mM K-ATP, and 0.2 mM Na-GTP; pH adjusted to 7.3–7.4. Cells were clamped with a Multiclamp 700B controller and Multiclamp software (Molecular Devices). Data from patched cells was acquired using a Digidata 1440 interface (Molecular Devices) and pClamp software (Molecular Devices). Patched neurons equilibrated for ∼5 min following successful whole cell configuration. Access resistance was monitored throughout experiments and if it reached above 25 MΩ, or changed by >20%, recordings were discarded. Patched neurons were considered acceptably healthy with a resting membrane potential below –50 mV and an action potential overshoot greater than +10 mV. Excitability curves were generated by injecting progressively larger positive current at 50-pA increments from 0 to 450 pA at the highest level of stimulation and counting the number of spikes at each level. This was done before CNO exposure, and after 4–6 min of 10 μM CNO exposure. Spike curves were analyzed using Clampfit software (Molecular Devices).

### Statistical analysis

IBM SPSS 25.0 was used for data analysis. A repeated-measures ANOVA was used to examine responses per minute across acquisition sessions and test sessions. The rejection criterion was set at *p* < 0.05. Following a significant interaction, within-subjects comparisons (two-tailed paired-samples *t* tests) were performed to determine the source of the interaction. Effect size was calculated as Cohen’s *d* for all significant effects (Table 1; Cohen, 1988; Rosenthal, 1994).

**Table 1.** Data structure, type of test used to analyze the data, and observed power of key results

	Data structure	Type of test	Power (Cohen’s *d*)
a	Normal distribution	Repeated-measures ANOVA	Main effect session: 1.784
b	Normal distribution	Repeated-measures ANOVA	Interaction (drug × vector): 0.247
c	Normal distribution	Paired samples *t* test	Main effect drug: 0.745
d	Normal distribution	Paired samples *t* test	Not significant
e	Normal distribution	Repeated-measures ANOVA	Main effect CNO: 0.388 Interaction (drug × current): 0.262
f	Normal distribution	Paired samples *t* tests	Main effects current: 200 pA: 1.204 250 pA: 3.095 300 pA: 2.807

## Results

Four rats (two DREADD, two GFP) were removed before analysis: one rat did not acquire the lever-press response, two rats had a viral vector infusion site dorsal to the PL, and one rat had extensive cannula-related damage to the DMS (for further explanation, see below, Histology). This left 10 rats in each group.

### Acquisition

**Figure 1.**
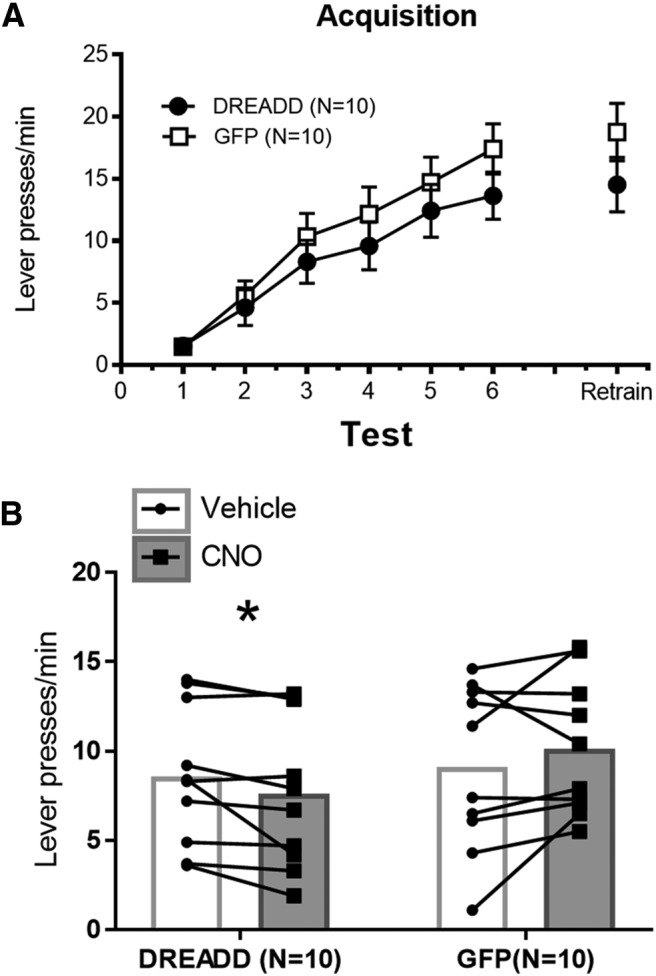
***A***, Acquisition of lever-press response over six training sessions with one retraining session in between the two test sessions. Mean ± SEM. ***B***, Test session results for rats that had received the DREADD or control (GFP) construct in the PL. CNO or vehicle was infused into the aDMS before the first test session, and rats received the opposite infusate before the second test session. Order of infusion was counterbalanced in each group; *p < 0.05.

All rats increased responding (lever presses/min) across training sessions, indicating successful learning of the operant response (Fig. 1*A*). A two (vector: DREADD vs GFP) × six (session) repeated-measures ANOVA yielded a main effect of session, *F*_(5,90)_ = 56.18, MSE = 9.78, *p* < 0.001^a^, but no main effect of vector or a vector × session interaction (*F*s < 1).

### Test

Inactivation of the PL-aDMS pathway attenuated the expression of operant responding during the test (Fig. 1*B*). A two (vector: DREADD vs GFP) × two (drug: CNO vs vehicle) repeated-measures ANOVA yielded a significant vector × drug interaction, *F*_(1,18)_ = 5.08, MSE = 1.95, *p* = 0.04^b^. Follow-up paired-samples *t* tests compared lever-press responding after CNO versus vehicle for each vector group separately. The DREADD group showed an attenuation of responding when tested with a CNO infusion, *t*_(9)_ = 2.36, *p* = 0.04^c^. In contrast, the rats that had received the GFP vector showed no difference in responding following CNO versus vehicle infusions into the DMS, *t*_(9)_ = 1.31, *p* = 0.22^d^. The pattern indicates that intra-DMS CNO effects were selective to the rats that had received PL DREADD transfection.

### Histology

**Figure 2.**
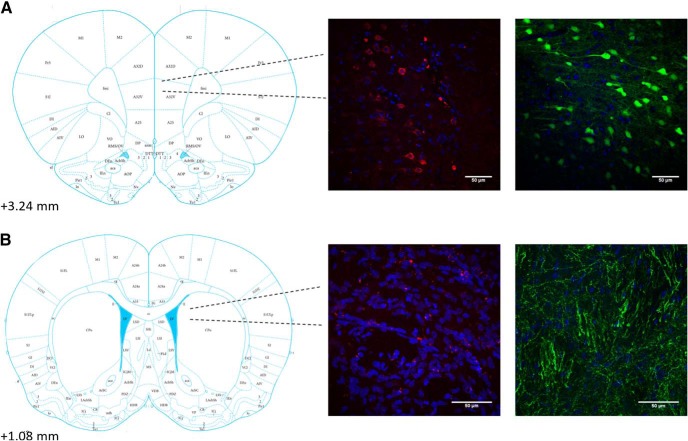
***A***, Location (left panel) and representative images (right panels) of cell bodies expressing DREADDs-mCherry construct (left) or GFP control construct (right) in the PL (area 32) at 40×. Blue is DAPI nuclear stain. ***B***, Location (left panel) and representative images (right panels) of axon terminals in aDMS expressing DREADDs-mCherry (left) or GFP (right) at 60×. Scale bars = 50 μm.

**Figure 3.**
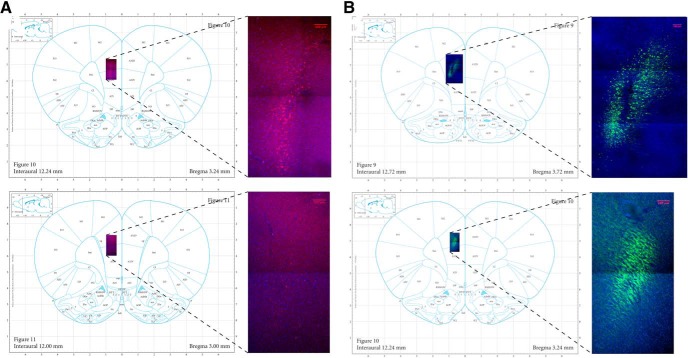
Location and representative DREADDs-mCherry (***A***) and GFP (***B***) spread in the PL (area 32) at 20×. Top panels are the infusion site and bottom panels are estimated posterior-most spread. Stitching was done with a 3% overlap in the images and each frame within the final image was 2048 × 2048 pixels. Scale bars = 100 μm.

**Figure 4.**
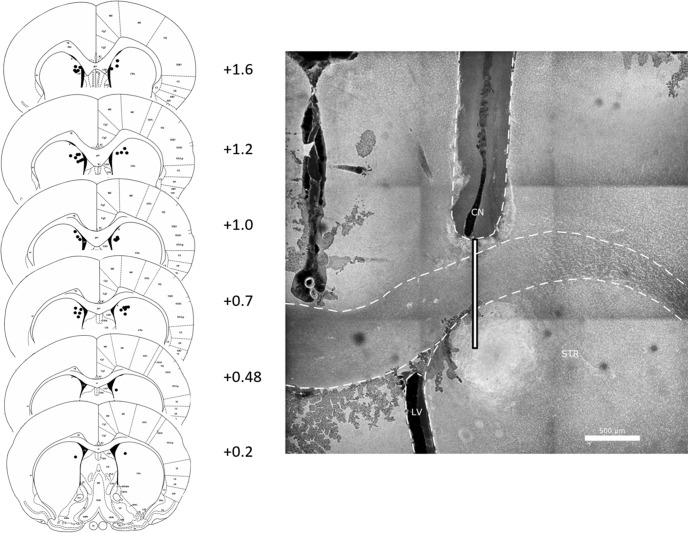
Left panel, Infusion sites in aDMS. Numbers are anterior-posterior distance in mm from bregma. Right panel, Example cannula placement. Shown is the right hemisphere, and guide cannula (CN) track. Guide cannula tips were above the DMS. The thin white bar shows where the infusion cannula (which protruded 1 mm below the tip of the guide cannula) was. STR, striatum; LV, lateral ventricle. Scale bar = 500 μm.

DREADD-mCherry expression and control GFP expression were verified in the cell bodies of the PL and axon terminals of the DMS in all rats. Examples are shown in Figure 2. Examples are also shown of typical dorsal-ventral and posterior-most spread from the PL infusion site (Fig. 3). Two rats were removed because the viral-vector infusion site in the PL was too shallow. Cannula placements in the DMS were also verified (Fig. 4). No rats had to be excluded from analysis for incorrect cannula placement, though one brain showed extensive damage from a cannula (possibly from infection) that affected tissue well beyond the cannula tract and DMS. This rat was excluded from analysis. Thus, three rats were removed during verification of viral expression, leaving the DREADD group with a final *n* = 10 and the GFP group with a final *n* = 10.

### Electrophysiology

**Figure 5.**
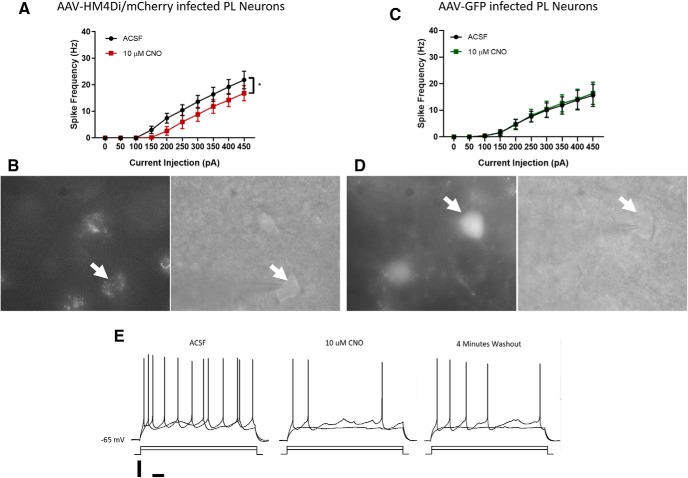
***A***, Excitability curve shows spikes elicited to progressively larger current injection of DREADDs-mCherry-expressing PL pyramidal cells before and after CNO (10 μM) exposure. ***B***, Example image of DREADDs-mCherry-expressing PL pyramidal cell in fluorescent (left) and infrared (right). ***C***, Excitability curve shows spikes elicited to progressively larger current injection of GFP-expressing PL pyramidal cells before and after CNO (10 μM) exposure. ***D***, Example image of GFP-expressing PL pyramidal cell in fluorescent (left) and infrared (right). ***E***, Example trace of DREADDs-expressing PL neuron; 4 min of CNO exposure caused a reduction in spike frequency to current injection compared to baseline, while removal of CNO from the bath caused a partial recovery of spike frequency. Scale bars = 20 mV and 100 ms, and stimulation was 250 and 350 pA for 1 s.

To confirm the effect of CNO on DREADDs-expressing PL pyramidal neurons, we used whole-cell patch-clamp electrophysiology to compare spike activity (number of spikes to 10 current steps, 0–450 pA) before and after CNO exposure (Fig. 5). DREADDs-expressing PL neurons showed fewer spikes after CNO exposure. In contrast, non-DREADDs expressing PL neurons spiked slightly more after CNO exposure, possibly because of CNO suppression of nearby DREADDs-expressing inhibitory interneurons.

A two (drug: CNO vs vehicle) × 10 (current: 0–450 pA) repeated-measures ANOVA on DREADDs-expressing PL neurons revealed a significant main effect of CNO on neuron spiking, *F*_(1,4)_ = 7.83, MSE = 31.49, *p* = 0.049^e^, and a significant drug × current interaction, *F*_(9,36)_ = 4.52, MSE = 2.82, *p* = 0.001^e^. Follow-up paired-samples *t* tests comparing CNO versus vehicle at each current step revealed significantly fewer spikes with CNO at current steps of 200, 250, and 300 pA (*p*s < 0.046^f^; Fig. 5*A*). The same analyses on GFP-expressing PL neurons revealed no CNO or drug × current interaction effects, *p*s > 0.45 (Fig. 5*C*).

## Discussion

The present results suggest that PL projections to a relatively anterior region of the DMS are involved in the expression of operant responding. This finding expands on the work by Trask et al. (2017) that had found involvement of the PL in expression of operant responding in the same paradigm, as well as that of Hart et al. (2018b), who demonstrated a role for PL-to-pDMS projections in the acquisition of goal-directed operant responding. The current results contrastingly show that a PL-to-a more aDMS pathway is important in the expression of operant responding early in training. This is unlikely to be a motor-related effect, given that studies have demonstrated that pharmacological inactivation of the PL (and therefore all of its projections) reduces only minimally-trained responding, and only in the acquisition context, while leaving other responses unaffected (Killcross and Coutureau, 2003; Trask et al., 2017; Shipman et al., 2018). Finally, we confirmed with *ex vivo* patch-clamp electrophysiology that cells in layer 5 of the PL expressing the DREADD-mCherry construct reporter showed attenuated spiking in the presence of CNO. Spiking of PL neurons expressing only the control GFP reporter was unaffected by CNO.

Although statistically significant, the size of the reduction in responding was numerically small in our DREADDs-expressing rats. However, there are several important points to keep in mind. First, we inactivated only a subset of projections from the PL to the aDMS, and the inactivation was probably less than total, as suggested by our electrophysiology results. Second, it is likely that other PL projections, besides just those to the aDMS, are important in expression of minimally-trained operating responding in the acquisition context; indeed, others (Trask et al., 2017) have shown a fairly large attenuation of responding with pharmacological inactivation of PL, which would inactivate all PL projections. Finally, it is worth comparing the magnitude of our effects to those of Hart et al. (2018b), who used a dual-vector approach and intraperitoneal injections of CNO during acquisition to silence PL-pDMS projections. Hart et al. (2018b) reported that in a 5-min choice (still-valued R2 vs devalued R1) test session, DREADDs-expressing rats that had received vehicle injection before acquisition sessions emitted an average of ∼18 R2 lever-presses; a separate group of DREADDs-expressing rats that had received CNO injection before acquisition sessions emitted an average of ∼12 R2 lever-presses. This translates to a reduction of approximately one lever-press per minute. We found that during a 10-min test session, vehicle-infused DREADDs-expressing rats emitted an average of ∼86 responses while CNO-infused DREADDs-expressing rats emitted an average of ∼76 responses. This also translates to a reduction of one lever-press per minute. Thus, despite a difference in methods and DMS terminal locations, the magnitude of operant response reduction as a result of DREADDs-mediated inactivation of PL-DMS terminals was similar in Hart et al. (2018b) and our experiment.

A recent concern with the use of DREADDs is that CNO does not appear to cross the blood-brain barrier; instead, the effects of systemic injections of CNO may be via the CNO metabolite clozapine, which binds with high affinity to DREADDs and binds with endogenous receptors (Gomez et al., 2017). We avoided this issue here by using intracranial CNO infusions. However, there may still be off-target effects caused by the use of a relatively high concentration of CNO in this method (Gomez et al., 2017). Therefore, we included two control procedures: (1) a group of rats that did not express DREADDs and (2) all rats received CNO and vehicle, in separate tests. Thus, we controlled for CNO effects as well as for potential vector effects. We also note here that an additional caveat to circuit-specific manipulation using DREADDs is that it may be difficult to completely isolate a specific pathway. For example, collateral projections of projection neurons expressing DREADDs may also be activated/inactivated by CNO. However, it is unclear how likely this is given that CNO is infused directly into the DMS.

We verified that CNO reduced spiking in PL neurons expressing DREADDs-mCherry while having no effect on spiking in PL neurons expressing GFP. However, this leaves unaddressed the question of whether or not CNO reduced spiking in DMS neurons as a result of reducing spiking in PL neurons projecting to the DMS. It seems likely that it did: first, the most straightforward interpretation of our behavioral results is that intra-DMS CNO reduced activation of PL projections to the DMS, which in turn reduced DMS activation. It is unlikely that CNO affected DMS neurons directly, since rats that did not express DREADDs were unaffected by intra-DMS CNO. Second, Lichtenberg et al. (2017) co-expressed the same DREADDs-mCherry construct as we used, along with channelrhodopsin, in neurons in the orbitofrontal cortex (OFC) and patch-clamped neurons in the basolateral amygdala (BLA) that were nearby fluorescing OFC terminals. Excitatory postsynaptic currents in these BLA neurons, produced by optical activation of fluorescing OFC terminals, were reduced in the presence of CNO. This suggests that CNO does reduce axon terminal activity in this DREADDs-mCherry construct. In addition, the design of our behavioral experiment makes alternative explanations less likely.

Like Hart et al. (2018b), we examined a role for PL-to-DMS projections in minimally trained operant responding, though our methods differ on a few critical points. First, we only trained one response with one outcome. Hart et al., trained two lever-press responses, each with its own unique outcome, and both levers were available during (choice) testing. Second, we did not devalue our reinforcer; thus, we did not distinguish between goal-directed versus habitual behavior. Third, we examined the PL-DMS pathway in a more anterior portion of the DMS (e.g., guide cannula implanted at +1.0 mm AP from bregma in our study vs AAV-Cre infusion at –0.4 to –0.5 mm AP in Hart et al., 2018b), rather than the PL projections to pDMS regions that have been more frequently associated with acquisition of goal-directed behavior. Fourth, we examined expression of responding, rather than the acquisition of responding, by inactivating the PL-DMS pathway before test rather than before each acquisition session. Finally, we used a different means of pathway-specific chemogenetic inactivation, implanting cannulae into the DMS to inactivate PL axon terminals after AAV8-DREADD infusion into the PL. In contrast, Hart et al., used a dual-virus approach, infusing a Cre-dependent DREADD viral construct into the PL and a Cre recombinase viral construct into the pDMS, and then inactivating the PL-pDMS pathway with intraperitoneal injection of CNO. Overall, our findings complement those of Hart et al. (2018b) who showed that the PL-pDMS pathway is important for the acquisition of goal-directed behavior. We show here that the PL-aDMS pathway is important for expression of minimally-trained operant behavior.

Many of the studies investigating the role of the PL in operant behavior have additionally confirmed whether responding was goal-directed or habitual (Corbit and Balleine, 2003; Killcross and Coutureau, 2003; Ostlund and Balleine, 2005; Tran-Tu-Yen et al., 2009; Shipman et al., 2018). Behavior is considered goal-directed if it is sensitive to reinforcer devaluation, whereas habitual behavior is insensitive to reinforcer devaluation. Though we did not use reinforcer devaluation to examine if our behavior was goal-directed, it is reasonable to assume that our minimally-trained operant response was goal-directed, as habit typically develops across many training sessions (Dickinson, 1985). This is further supported by the findings of Shipman et al. (2018), who showed that the PL plays a transitory role in the development of operant responding: inactivation of PL reduced minimally-trained goal-directed instrumental behavior, but not more extensively-trained instrumental behavior that is goal-directed. The PL has never been linked to habit.

Despite dense anatomic connections from the PL to the aDMS, research has tended to focus on the pDMS in goal-directed behavior. The pDMS has been defined as the DMS beginning around +0.24 mm anterior to bregma (Hart and Balleine, 2017). The focus on the pDMS is largely based on an early study by Yin et al. (2005b). Yin et al. (2005b) found that pre-training or post-training lesions of the posterior region of the DMS impaired the acquisition and expression of goal-directed behavior (target posterior coordinates at –0.4 mm AP relative to bregma, compared to +1.0 mm AP in the current study). However, the effects of aDMS lesions were actually somewhat inconclusive, as pre-training aDMS lesions did not affect expression of goal-directed behavior at test but post-training aDMS lesions did. Other research has provided support for the idea that the pDMS, but not the aDMS, is important for goal-directed responding. For example, functional disconnection of the parafascicular thalamus and pDMS disrupts goal-directed responding, whereas disconnection of the parafascicular thalamus and aDMS has no effect (Bradfield et al., 2013).

Nonetheless, other studies have found that the aDMS, in addition to the pDMS, is important for goal-directed behavior. Corbit and Janak (2010) trained two different lever-press responses and then used satiation to devalue the outcome associated with one response. They found that temporary inactivation with baclofen/muscimol of either the aDMS or pDMS (coordinates at +1.2 and –0.3 mm AP relative to bregma, respectively) during acquisition resulted in insensitivity to outcome devaluation at time of test in an operant task (Corbit and Janak, 2010). This result suggests that aDMS and pDMS both seem to be involved in goal-directed responding. Further studies by this lab also showed a role for the aDMS in goal-directed behavior with an alcohol reinforcer (Corbit et al., 2012). Thus, there is some evidence for aDMS involvement in goal-directed behavior despite a literature that focuses largely on the pDMS.

In conclusion, we found that the PL-aDMS pathway is important in the expression of operant responding. Thus, we expand on previous research to show, using circuit-specific chemogenetic silencing, a role for a PL-to-aDMS pathway in the expression of operant behavior to complement the demonstrated role of a PL-to-pDMS pathway in the acquisition of operant behavior.

## Synthesis

Reviewing Editor: Yavin Shaham, NIDA-IRP/NIH Decisions are customarily a result of the Reviewing Editor and the peer reviewers coming together and discussing their recommendations until a consensus is reached. When revisions are invited, a fact-based synthesis statement explaining their decision and outlining what is needed to prepare a revision will be listed below. The following reviewer(s) agreed to reveal their identity: Morgan James.

The reviewers found merit in the study but have identified two major issues. The first is the modest scope of the paper in light of the recent similar paper from the Balleine lab. Addressing this issue with additional experiments is not mandatory for successful revision but expanding the scope of the study is encouraged. The second is the lack of sufficient evidence that the projection-specific inhibition method indeed inhibits neuronal activity in aDMS. We recommend adding such data in the revision. In addition to the reviewers' specific comments, please address the minor comments below.

1. Line 69: delete ‘for the first time’

2. Please use JN/eNeuro format of in-text citations (1st author et al) and reference section

REVIEWER 1

These data provide a nice characterization of the PL-anterior DMS pathway in the expression of an operant task. The inputs from PL to aDMS have not previously (to my knowledge) been examined in this context, and thus are interesting. I felt, however, that this initial finding could used as the basis of several other experiments designed to characterize the role of this projection in operant behaviors. For example - is this projection important for the acquisition of operant behavior? Does this projection become less important following the transition to 'habitual' responding? Is PL input to aDMS more/less/equally important for these behaviors compared to its inputs to pDMS?

Other points to consider:

1) It would be good to see the data from non-DREADD expressing PL neurons in Figure 2, especially given the trend towards increased firing post-CNO.

2) related to the above - did the authors consider recording from aDMS neurons pre and post CNO application? This would seem more relevant to the experimental design.

3) A photomicrograph to accompany Fig 1c would be useful.

4) Some discussion of what is considered anterior vs posterior DMS would be useful (with reference to Hart 2018)

REVIEWER 2

The authors present a study where they investigate the role of the PL-->aDMS pathway in the expression of an operant response. The rats were trained to press a lever for food reinforcement in 6 sessions, and they were subsequently tested in extinction with micro-injection of CNO in aDMS. They found that this manipulation caused a small but statistically significant reduction in lever presses. While I think the study was conducted well, me feeling is that these results are still too preliminary. Furthermore, the I feel that the methodology is not sufficiently validated.

Strengths

The potential finding that activity in the PL-->aDMS pathway contributes to operant responding is novel, and of interest to the field.

The paper is well written, clear, and scholarly. The results are clear, and the statistics used are appropriate. The discussion does a great job of integrating these findings within the broader literature, while taking into account limitations.

Major weakness

My main concern is the extent to which the PFC-->aDMS pathway was actually manipulated in this study. Unfortunately, the effect of CNO in the terminals in aDMS was not validated. While this is challenging, it is possible to show that activation of hM4Di in the terminals can reduce excitability. In addition to this, the included figure (1E, Left) shows what can only be considered very weak expression of the DREADD terminals in the aDMS. So I am left with the impression that the extent to which the manipulation has worked is an open question.

Comments

While the cannula locations are well described, it will be helpful to additionally show the range of DREADD expression in mPFC. In the current version, this is not displayed. I don't think quantifying number of cells is helpful, but displaying the ventral/dorsal extent, as well as the anterior/posterior extent will allow the reader to understand what was manipulated.

## Author Response

We would like to thank the two reviewers and the editor for the time they spent reviewing our manuscript and for their comments. We believe this revised manuscript is improved because of their work. In addition to addressing their comments, we have also added additional details on the two AddGene viral constructs (p. 5). Revisions are indicated in red font.

Editor

1. Modest scope of the paper in light of the recent similar paper from the Balleine lab. Addressing this issue with additional experiments is not mandatory for a successful revision but expanding the scope of the study is encouraged.

We envision this manuscript as a brief communication. Our finding of a role for prelimbic cortex projections to the anterior dorsomedial striatum in expression of instrumental conditioning is novel. In addition, we believe that we used a strong experimental design (within-subjects comparison of CNO vs. vehicle; inclusion of non-DREADDs rats) that gives us confidence in the result. Additional confidence comes from the results of our patch-clamp experiments showing that only DREADDs-expressing prelimbic cortex neurons are modulated by CNO and not GFP-expressing prelimbic cortex neurons.

2. Lack of sufficient evidence that the projection-specific inhibition method indeed inhibits neuronal activity in aDMS. We recommend adding such data in the revision.

We have added a paragraph to the discussion addressing this issue (p. 16). For the construct that we used (AAV8-hSyn-hM4Di-mCherry), verification of terminal inhibition by CNO has been done quite elegantly by Wassum and colleagues (Lichtenberg et al., 2017). For behavioral experiments, these researchers used the same DREADDs-mCherry construct that we did and also like us, infused CNO into a target brain region. For electrophysiology, these researchers infused the same DREADDs-mCherry construct, along with channelrhodopsin, into one brain region and performed whole-cell patch-clamp on postsynaptic neurons in “highly fluorescent regions” in a target brain region. Optical activation of these highly fluorescent terminal regions induced excitatory postsynaptic currents in postsynaptic neurons in the target brain region. These EPSCs were significantly reduced in the presence of CNO.

3. Line 69: delete ‘for the first time’

Done.

4. Please use JN/eNeuro format of in-text citations (1st author et al) and reference section

Done.

Reviewer 1

1. This initial finding could be used as the basis of several other experiments designed to characterize the role of this projection in operant behaviors. For example - is this projection important for the acquisition of operant behavior? Does this projection become less important following the transition to ‘habitual’ responding? Is PL input to aDMS more/less/equally important for these behaviors compared to its inputs to pDMS?

These are all questions that are of interest to us. We envision the current manuscript as a “brief communication” reporting an initial, novel finding that we believe is of interest to the field.

2. It would be good to see the data from non-DREADD expressing PL neurons in Figure 2, especially given the trend towards increased firing post-CNO.

We collected additional data to address this point. A limitation of the non-DREADDs expressing PL neuron data that we reported in the initial submission is that it was collected from non-DREADDs expressing PL neurons in slices that also contained DREADDs-expressing PL neurons. This complicates interpretation because CNO may have indirect effects on non-DREADD expressing PL neurons through modulation of DREADD-expressing neurons (e.g., inhibitory interneurons). Therefore, we collected a new set of data from slices that expressed only our control construct, GFP, the same as our control rats in the behavioral experiment. Thus, in Figure 5, we now report the results of recordings from GFP-expressing (control) PL neurons, in addition to DREADDs-mCherry PL neurons. We did not see any change in spiking in GFP-expressing neurons in response to CNO.

3. Did the authors consider recording from aDMS neurons pre and post CNO application? This would seem more relevant to the experimental design.

See response to comment #2 of the Editor.

4. A photomicrograph to accompany Fig 1c would be useful.

This has been incorporated into a new Figure 4.

5. Some discussion of what is considered anterior vs posterior DMS would be useful (with reference to Hart 2018)

We have added a sentence (p. 18) about Hart and Balleine's distinction: “The pDMS has been defined as the DMS beginning around +0.24 mm anterior to bregma (Hart & Balleine, 2017).”

Reviewer 2

1. While I think the study was conducted well, my feeling is that these results are still too preliminary.

See response to comment #1 of Reviewer 1.

2. My main concern is the extent to which the PFC-->aDMS pathway was actually manipulated in this study. Unfortunately, the effect of CNO in the terminals in aDMS was not validated. While this is challenging, it is possible to show that activation of hM4Di in the terminals can reduce excitability.

See response to comment #2 of the Editor.

3. The included figure (1E, Left) shows what can only be considered very weak expression of the DREADD terminals in the aDMS.

Our infusions in the PL were very circumscribed (see new Figure 3), which is likely why we did not see widespread expression in the aDMS. Despite this, we were able to observe a suppression of instrumental responding with intra-DMS CNO infusion only in rats that had received intra-PL infusion of the DREADDs construct. We believe that the strength of our experimental design, with both a within-subject control (vehicle infusion) and a between-subject control (expression of a non-DREADD construct) lends confidence to our results.

4. While the cannula locations are well described, it will be helpful to additionally show the range of DREADD expression in mPFC. In the current version, this is not displayed. I don't think quantifying number of cells is helpful, but displaying the ventral/dorsal extent, as well as the anterior/posterior extent will allow the reader to understand what was manipulated.

This has been added (see new Figure 3). We have also used the newest Paxinos and Watson (2014) atlas to display estimated PL location (at the border of dorsal/ventral area 32; area 32).
